# Sequence Alignment Tools: One Parallel Pattern to Rule Them All?

**DOI:** 10.1155/2014/539410

**Published:** 2014-07-24

**Authors:** Claudia Misale, Giulio Ferrero, Massimo Torquati, Marco Aldinucci

**Affiliations:** ^1^Computer Science Department, University of Turin, Italy; ^2^School of Life and Health Sciences, University of Turin, Italy; ^3^Computer Science Department, University of Pisa, Italy

## Abstract

In this paper, we advocate high-level programming methodology for next generation sequencers (NGS) alignment tools for both productivity and absolute performance. We analyse the problem of parallel alignment and review the parallelisation strategies of the most popular alignment tools, which can all be abstracted to a single parallel paradigm. We compare these tools to their porting onto the FastFlow pattern-based programming framework, which provides programmers with high-level parallel patterns. By using a high-level approach, programmers are liberated from all complex aspects of parallel programming, such as synchronisation protocols, and task scheduling, gaining more possibility for seamless performance tuning. In this work, we show some use cases in which, by using a high-level approach for parallelising NGS tools, it is possible to obtain comparable or even better absolute performance for all used datasets.

## 1. Introduction

Next generation sequencers (NGS) have increased the amount of data obtainable by genome sequencing; a NGS run produces millions of short sequences, called* reads*, that is, a sequence of nucleotides containing also information about the* quality* of the sequencing process, which determine the reliability of the nucleotide called during sequencing. The alignment process maps reads onto a reference genome in order to study the structure and functionalities of sequenced data.

The rapid evolution of sequencing technologies, each one producing different datasets, is boosting the design of new alignment tools. Some of them target specific datasets (e.g., short reads, long reads, and high-quality reads) or even data from specific sequencing technologies. Since the alignment process is computationally intensive, many alignment tools are designed as parallel applications, typically targeting multicore platforms. Several of them are based on the well-known Smith-Waterman algorithm, which is known to be computationally expensive. For this reason, many of them are already parallel, typically leveraging on multithreading. Also in some cases, SIMD parallelism (via either SSE or GPGPU) is also exploited.

Due to specialisation, some of these tools provide the users with superior alignment quality and/or performance. With the ever-growing number of sequencing technologies, it can be expected that the scenario of specialised alignment tools will widen yet more.

Although the market of NGS alignment tools is growing, to date, the parallel programming methodologies used to design these tools do not embrace much more than low-level synchronisation primitives, such as mutual exclusion and atomic operations. In the hierarchy of abstractions, it is only slightly above toggling absolute binary into the front panel of the machine. In the NGS community, programming multicore for performance is still perceived according to “the closet to the metal the fastest”, thus exclusively focusing on extreme optimisation of the code for a single algorithm and a single platform. We believe that correctness, productivity, time-to-market, and porting of existing legacy codes are equally important targets.

In this paper, we advocate high-level programming methodology for NGS alignment tools for both productivity and absolute performance. We analyse the problem of parallel alignment and review the parallelisation strategies of some of the most popular alignment tools (such as Bowtie and BWA), which can all be abstracted by the* farm* paradigm (a.k.a. master-worker or task-*farm*) [1, 2]. We compare these tools to their porting onto the FastFlow pattern-based programming framework [3], which provides the master-worker as high-level pattern liberating the programmers from all synchronisation details but providing them with seamless performance tuning and eventually equal or better absolute performance on tested tools.

This paper is organised as follows.  Section 2 presents related work on alignment tool performance, optimisations, and benchmarking, while in Section 3 the FastFlow library for high-level parallel programming models is presented. Our case studies, Bowtie and BWA, are also presented in Sections 3.3.1 and 3.3.2, respectively.  Section 4 provides a dataset analysis, which reports alignment rates of Bowtie2, BWA, and BLASR on corrected and uncorrected PacBio datasets. In Section 5, performance evaluations on short reads datasets (20–200 bp) with Bowtie2 and the comparison with our implementation with respect to the new release of the software are presented. Performances on PacBio dataset alignments executed with Bowtie2, BWA-MEM, and BLASR are also discussed.  Section 6 concludes the paper.

## 2. Related Works

Many algorithms for sequence alignment have been proposed and different tools were implemented that entirely exploit multithreading on homogeneous and heterogeneous platforms.

### 2.1. Alignment Tools

The first step done before an alignment is to create and load the reference genome. The used techniques are hash tables and Burrows-Wheeler transform [4]. The hash-based technique builds a hash table for subsequences of both genome and reads. Keys are created by hashing subsequences and values are lists of positions in which subsequences can be found. Hash-based tools, such as SOAP [5] or SHRiMP [6], are particularly suitable for short sequences alignment. The Burrows-Wheeler transform (BWT) [4] is a string permutation algorithm used in data compression tools as bzip2. Ferragina and Manzini have enhanced it with the implementation of the FM-index [7, 8], an opportunistic data structure for text compression that permits fast substring queries. Bowtie [9] and Bowtie2 [10] are Burrows-Wheeler transform (BWT) based tools. Bowtie2 aligns longer reads and supports gapped, local, and paired-end alignment modes. More details on Bowtie2 are described in Section 3.3.1. MrFAST [11] maps short reads emphasising the discovery of structural variation and segmental duplications. It is possible to map both single-end and paired-end reads and to support up to 4+4 base-pair indels. BWA [12] and SOAP2 [13] use the FM index in order to create a suffix array on sequences compressed by the BWT algorithm. The combination of these two algorithms permits the creation of a compressed genome that can be fully loaded in memory. This technique has the limitation of a lower sensitivity in alignment with respect to hash-based indexing and of a reduction of maximum allowed mismatches (for instance, Bowtie2 allows only up to one mismatch) but has the advantage to make the alignment faster. Burrows-Wheeler aligner (BWA) consists of three algorithms: BWA-backtrack, BWA-SW, and BWA-MEM. The first is designed for reads up to 100 bp, while the other two for longer sequences. BWA-MEM is used typically for high-quality reads, having better performance than BWA-backtrack for 70–100 bp reads. BLASR (Basic Local Alignment with Successive Refinement) is a Single Molecule Sequencing alignment tool specifically targeting PacBio datasets, where divergence between the read and genome is dominated by insertion and deletion errors [14]. This tool has been benchmarked with both real and synthetic datasets produced by the PacBioRS instrument and results obtained show that it is possible to map SMS reads with high accuracy and speed.

### 2.2. Tools Parallelisation and Optimisations

Alignment tools, generally, exploit parallelism via multithreading. As an example, Bowtie, both versions Bowtie1 and Bowtie2, implements multithreading with Posix Threads. The BWA aligner is implemented in two different versions: multithreads by exploiting a pool of Posix Threads in BWA and by using MPI (message passing interface) to exploit both shared memory and distributed clusters (pBWA [15]). Posix Threads is also exploited by the BLASR aligner for multithreading. SHRiMP and SHRiMP2 are parallel alignment tools, available also in a distributed version [16], implemented upon the MapReduce programming model [17]. Alignment tools that exploit GPUs were also presented. An example is SOAP3 [18], the GPU-based version of SOAP2. SOAP3 is at least 7.5 to 20 times faster than BWA and Bowtie, respectively. In addition, BarraCUDA [19] and CUSHAW [20] are short reads alignment tools that exploit GPUs.

### 2.3. Datasets

There exist several technologies for DNA sequencing, which produce reads of different lengths. At today, the most popular sequencing platforms for long read generation are the Roche 454 or Ion Torrent PGM platforms, whereas for short read generation are the Illumina and Applied Biosystems platforms. Novel sequencing platforms, such as SMRT (Single Molecule Real Time sequencing) PacBio RS family [21] by Pacific Biosciences, generate reads with a mean length of 8.500 bases and longest reads exceeding 30 Kbp. From metagenomic studies to genome-based personalised patients care, longer reads are mandatory to solve structural complexities in nucleotide sequences that are analysed in heterogeneous assays including* de novo* genome assembly [22], haplotype phasing [23], transcriptome analysis [24], and structural and copy number analysis [25]. In [26], a review of alignment algorithms, by introducing their practical applications on different types of experimental data, is proposed. Authors state that short-read alignment is no longer a bottleneck and consider future development of alignment algorithms with respect to emerging long sequence reads. In fact, the new trend in sequencing technologies is to generate datasets with longer sequences with respect to Roche 454 GS FLX Titanium and Ion Torrent PGM, which generate reads with length up to 1000 bp by the former and up to 200 by the latter. PacBio is the new sequencing technology producing reads which length can reach 35 Kbp, having an error rate around 15% that is uniform within sequences. In [27], authors developed a simulation and evaluation suite, SEAL, which simulates NGS runs for different configurations of various factors, including sequencing error, indels, and coverage. They propose also criteria to compare the performance of alignment tools and evaluated Bowtie, BWA, mr- and mrsFAST, Novoalign, SHRiMP, and SOAPv2, considering accuracy and runtime. An evaluation of alignment algorithms for RNA-Seq is proposed in [28], where they evaluated 14 widely used alignment programs with respect to three different algorithmic classes: hashing of the reference transcriptome, hashing of reads, and compressed FM-index. They focused on precision, recall, and performance for different read lengths and on numbers of mismatches and indels within a read. These studies evidenced how each algorithm is characterized by peculiar overall performances and tolerance to errors in the sequences. This latter parameter became important as length of analysed reads increased. In fact, in [29], authors show an algorithm and associated software tool, PBJelly, a pipeline for gap filling and genome improvement that aligns long sequence reads to draft assembles in order to close or improve captured gaps. Results provide a 24% mapped coverage of PacBio long reads and a 99% of addressed gaps, with the closure of the 69% and the 12% improvement of all gaps in genomic data from* Drosophila pseudoobscura*.

## 3. Materials and Methods

### 3.1. High-Level Parallel Programming Models: The FastFlow Framework

Multicore platforms are* de-facto* small-scale on-chip parallel computer. The only way to increase performance on a multicore is by exploiting thread-level parallelism. Parallel programs are inherently more difficult to write than sequential ones, because concurrency introduces several new problems that programmers should take into account carefully. Developers are then facing the challenge of achieving a trade-off between high-end performance and time to solution in developing applications on multicore platforms in which the number of cores per CPU is increasing.

Parallel software engineering addressed this challenge via high-level sequential language extensions, parallel coding patterns, and algorithmic skeletons aimed at simplifying the porting of sequential codes to parallel architecture while guaranteeing the efficient exploitation of concurrency [30, 31].

Parallel design patterns have been recognised to have the potential to induce a radical change in the parallel programming scenario, such that new parallel program will be capable of exploiting the high parallelism provided by hardware vendors [32]. Parallel design patterns make programmer tasks simpler and the whole application development process more efficient. They provide tested and efficient parallelism exploitation patterns as compositional building blocks, without the need of implementing, tuning, and maintaining ad hoc solutions. A higher level of abstraction, with respect to more traditional HPC approach, is thus offered to the programmer.

In this respect, the FastFlow parallel programming framework [3], offers an important methodological approach that allows applications to be easily parallelized on a variety of multi/many-core platforms. Also, thanks to its efficient lock-free run-time support [33], applications developed on top of FastFlow typically exhibit a good speedup with a minimal tuning effort.

**Figure 1:**
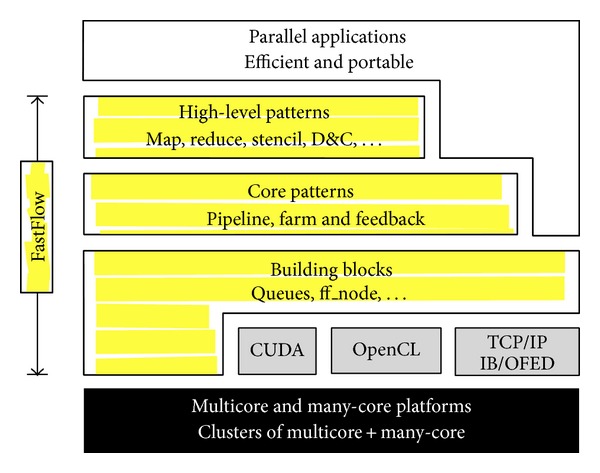
FastFlow Layered Design.

FastFlow is a parallel framework targeting shared memory multi/many-core and heterogeneous distributed systems. It is implemented in C++ on top of the Posix Threads (Pthreads) library and provides developers with a number of efficient parallel patterns [3]. As shown in Figure 1, it is designed as a stack of layers in order to abstract the level of parallelism, starting from core level up to high-level programming construct (such as map, reduce, stencil or Divide & Conquer). The abstraction process has two main goals: (1) to promote high-level, platform-independent parallel programming, and in particular skeletal programming (i.e., pattern-based explicit parallel programming), and (2) to promote efficient programming of applications for both homogeneous and heterogeneous platforms and clusters of them.

FastFlow has been used to design a variety of parallel algorithms, including Smith-Waterman [34], C4.5 data classifier [35], and Gillespie simulators for systems biology [36].

### 3.2. The Paradigmatic Structure of Parallel Alignment Tools

As discussed in related works (Section 2), a plethora of sequence alignment tools is currently available. Some of them target specific datasets (e.g., short reads, long reads, and high-quality reads) or even specific sequencing technologies (e.g., BLASR for PacBio). Several of them are based on the well-known Smith-Waterman algorithm, which is known to be computationally expensive; thus, many of them have already a parallel implementation typically exploiting multithreading. In some cases, multithreading is coupled with SIMD parallelism to make use of hardware accelerators, either processor Streaming SIMD Extensions unit (SSE) or General-Purpose Graphics Processing Units (GPGPUs). They are typically employed in the mapping of a single read.

Thanks to specialisation, some of these tools might provide the users with superior alignment quality and/or performance. It is of particular interest to identify and engineer the building blocks needed to develop a parallel alignment tool that is at the same time efficient and portable. Ideally, such building blocks can provide any forthcoming alignment tool with absolute performance, performance portability, and reduced development time.

Indeed, the parallelisation of sequence alignment problem exhibits a number of distinguished features.

- There exists one (or a set of, in the future,) reference sequences (e.g., genome). The single sequence is typically read-only data.
- There exists a set of reads to be aligned against the reference. It is also read-only data. The specific attributes of the reads (e.g., length, quality) depend on the dataset. They can anyway be independently aligned against the reference(s). With the growing size of the dataset, they are likely to be available as a stream of data flowing from a permanent storage.
- The assembly of results from independent alignment frequently does not require a complex merging operation. In case a merging phase is required (e.g., to provide a global filtering of the data), it is expected to be an online process on the result stream flowing to a permanent storage.

These features fit into the master-worker parallel paradigm (i.e., a variant of the farm paradigm), or the more general composition of pipeline and farm paradigms in the case the process requires complex a merging operation (e.g., ordered merging). As a matter of fact, all the most popular parallel alignment tools, including Bowtie2, BWA, and BLASR, implement a master-worker paradigm, where each worker cycles over the following three steps:

1. gets a sequence to align from the shared input file;
2. aligns the read against the genome loaded into the shared index file;
3. populates shared data structures with results and statistics.

During the first and last steps, shared data structures are accessed in a read/write mode. These accesses are regulated via mutual exclusion (either blocking lock or atomic-based spin-lock, depending on the configuration). Furthermore, during these steps, the memory space to accommodate reads is dynamically allocated and deallocated, which might induce further mutual exclusion operations within the memory allocator. Each worker thread iteratively gets a single read from the input dataset and maps it onto the reference genome. This behaviour, usually named on-demand scheduling, enforces load balancing among worker threads.

Interestingly enough, all of them are developed with extremely low-level programming tools, such as spin-lock and atomic operations. They might provide the applications with low-overhead synchronizations but certainly make them hardly portable across different platforms and operating systems. Furthermore, such low level optimizations require nontrivial debugging and a large performance-tuning effort.

As shown in the next section, the adoption of an engineered master-worker pattern simplifies the work, guarantees the portability of the application, and provides the application with good performance. This adoption has been applied to two aligners, Bowtie2 and BWA-MEM. To definitely assert that the proposed pattern is the best for every aligner, we should test it on each tool. It is difficult because of their constant increasing number, but we can say that, for its nature, this pattern helps on simplifying both the parallelisation process and further optimisations.

### 3.3. Case Studies

#### 3.3.1. Bowtie

The Bowtie2 (a.k.a. Bowtie version 2) alignment tool can align reads of very different length. The human genome loading requires a fairly limited amount of memory (about 2.3 GB) and it makes the tool usable from both workstations and laptops. The original source code of Bowtie2 implements parallelism by using the Posix Threads library according to a master-worker pattern.

Each worker iteratively cycles the three steps described in Section 3.2.

In order to asses expressiveness and efficiency of the pattern-based approach, Bowtie2 (version 2.0.6) has been ported on top of the FastFlow library (*Bowtie2-FF*) [3, 37]. The porting basically consisted in substituting the low-level task dispatching code with an instance of the* farm* pattern (i.e., a C++ factory object), specialised in the variant master-worker. Overall, this required to alter less than two dozens of code lines out of about 40 K code lines (excluding comments) of the whole Bowtie2 source code.

**Figure 2:**
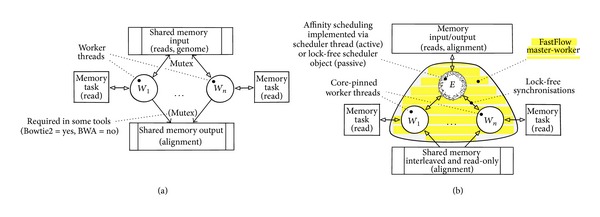
Typical thread orchestration in parallel alignment tools. (a) Low-level design (e.g., Bowtie2, BWA); (b) pattern-based design with FastFlow (e.g., Bowtie2-FF, BWA-FF).

The synchronisation schemas of both original Bowtie2 and Bowtie2-FF are shown in Figure 2. Observe that the two applications are almost identical both in the orchestration of parallel activities (i.e., master-worker paradigm) and in the business code (i.e., C++/SSE workers code, input and output code). Nevertheless, the usage of FastFlow master-worker pattern has several advantages with respect to low-level code as follows.

- Thread creation and synchronisation are trasparently made available by the parallel pattern. This simplifies the coding and enhance portability on different platforms and threading libraries (e.g., Windows native threads).
- Pattern run-time behaviour can be configured according to different scheduling policies (e.g., static, on-demand, and affinity) without changing the code.
- The lock-free run-time support minimises concurrency overhead due to coherency traffic, thus exhibiting a superior speedup on fine-grain and irregular workloads.

Also, the FastFlow framework offers the opportunity to easily couple thread pinning and memory affinity. As an example, in the Bowtie2-FF implementation, each worker private data structures have been allocated on the memory node connected to the core that is executing the worker pinned on it. This way it is possible to get the best memory access latency; that is, each worker thread needs less time to access to the memory and retrieve private data. In order to improve access to the genome, it has been allocated with an interleaved policy, that is, allocating memory pages into all memory nodes on the system (Round-Robin scheduling policy). This way it is possible to avoid memory hot spots on the access to the genome (concurrently accessed by many cores). To understand the gain breakdown of the different techniques, in Section 5.1, three variants of Bowtie2-FF have been tested:

1. Bowtie2-FF (bt-FF): master-worker with workload dynamically partitioned among workers;
2. Bowtie2-FF with thread pinning (bt-FF (pin)): master-worker with threads pinning on cores and memory affinity for private data;
3. Bowtie2-FF with thread pinning and genome interleaving (bt-FF (pin + int)): master-worker with threads pinning on cores, memory affinity for private data, and interleaved allocation policy among memory nodes for shared data (genome).

For further implementation details, please refer to [37].

Bowtie2-FF has been developed as a porting version 2.0.6 on the FastFlow library. In February 2014, the version 2.2.1 of Bowtie2 has been released. It has been improved in the index querying efficiency using “population count” instructions available since SSE4.2. In this set, the STTNI instructions (String and Text New Instructions) have been added, which contain several new operations for character searches and comparison on two 16 bytes operands. The two versions do not differ in the orchestration of threads.

#### 3.3.2. Burrows-Wheeler Aligner (BWA)

The BWA alignment suite includes three algorithms based on the suffix-array based representation of data (Burrows-Wheeler): BWA-backtrack, BWA-SW, and BWA-MEM [12, 38]. As previously mentioned, the first algorithm is designed for relatively short sequences (Illumina reads up to 100 bp), while the rest two for longer sequences ranged from 70 bp to 10 Kbp. Compared with Bowtie, it needs a slightly larger memory footprint (about 3.2 GB of memory [39]).

In all three variants, the BWA tool is designed according to a master-worker paradigm as described in Section 3.2 and Figure 2(a). They differ for the business code used to instantiate the workers threads.

As for Bowtie2, each worker of BWA iteratively cycles the three steps described in Section 3.2. Differently from Bowtie2, BWA prefetches all the reads from the storage before scheduling them to workers. This makes it possible to schedule them to workers with a single atomic increment operation that points to the next task to be executed in the array of tasks, which is also used to avoid mutual exclusion in the output of results. A specialised work-stealing mechanism is used for load-balancing.

Theoretically, the FastFlow master-worker implementation has still performance edge against the described implementation since it also avoids all coherency traffic due to atomic operation (thanks to the memory fence-free/atomic-free design of FastFlow run-time). However, this edge becomes evident only for very fine grain tasks (hundreds of clock cycles), whereas typical task grain in BWA is orders or magnitudes larger. Still, master-worker pattern simplifies the design because it implements a transparent dynamic load-balancing strategy and does not require any ad hoc rebalancing strategy.

## 4. Dataset Analysis

In this section, datasets used to compare performances between Bowtie2 and Bowtie2-FF are firstly present (Section 4.1). Then, results about mapped and unmapped sequences of PacBio datasets by using Bowtie2, BWA, BWA-MEM-FF, and BLASR are shown (Section 4.2).

### 4.1. Roche 454 and Illumina Datasets

Within this work, we aligned datasets obtained with three different sequencing technologies in order to show how they behave with various lengths. More precisely, for our analysis on Bowtie2, we selected 4 short reads (SRR027963, SRR078586, SRR502198, and SRR341579) and 3 long reads experiments (SRR003161, Human-Ref19-1, and Human-Ref19-2). The formers report genomic sequences from CTCF ChIP-Seq experiments performed on IMR90 cell line (SRR078586), Exome sequencing from phase 1 of 1000 Genomes Project (SRR502198), and a dataset from Hi-C assay on K562 cells (SRR341579, SRR027963). For long read datasets, we choose three whole human genome sequencing, one from phase 2 of 1000 genome project (SRR003161).  Table 1 summarises used datasets with their characteristics and sequencing platform.

### 4.2. PacBio Human Datasets

**Figure 3:**
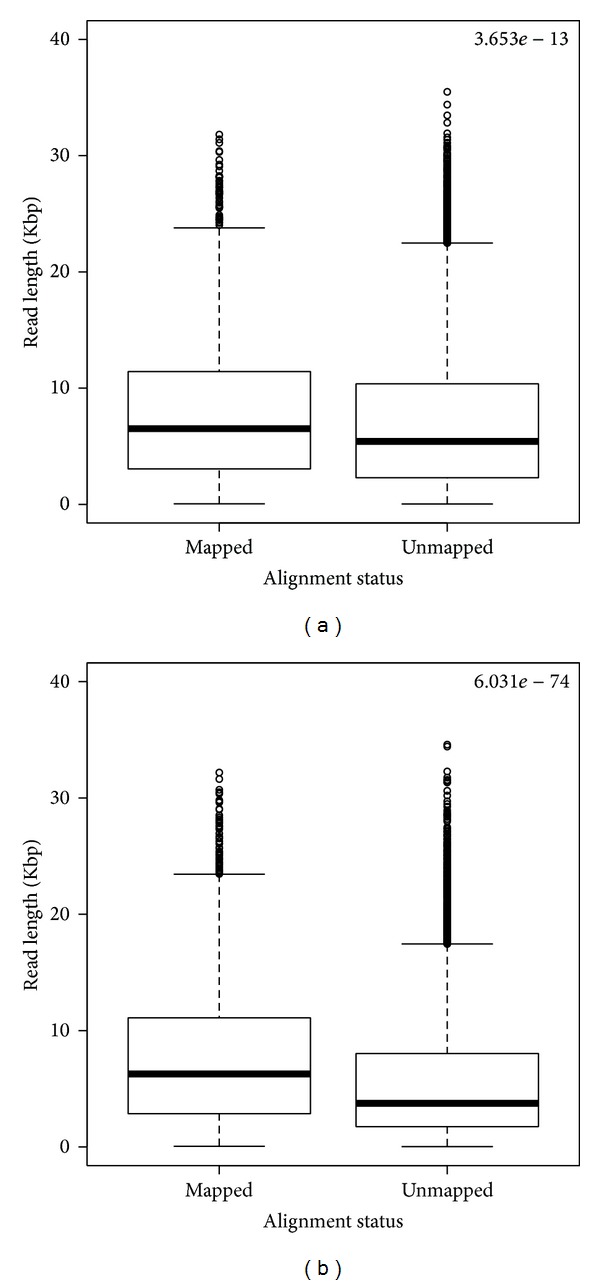
Length of mapped and unmapped reads for PacBio Human-Ref19-1 (a) and Human-Ref19-2 dataset (b) obtained with bt-FF. *P* value from two-tailed unpaired* t* test is reported on top.

We also selected a portion of two PacBio Bioscience PacBio RS II technologies (Human-Ref19-1, Human-Ref19-2). By comparing the length of mapped and unmapped reads, we observed that Bowtie2 was able to align reads longer than 10 Kbp (max length 35 Kbp). Despite expectations, majority of shorter reads (less than 3 Kbp) were not mapped by the algorithm (Figure 3). This is probably caused by the unusual sequencing error related to this technique, uniformly distributed along the reads [40]. Alignment percentages of both datasets are reported in Table 2. In these tables, we report the very low alignment rate of Bowtie2, which is at most about 13%. We focused on these subsets because of the low alignment rate on relatively short sequences, as reported in Figure 3.

**Figure 4:**
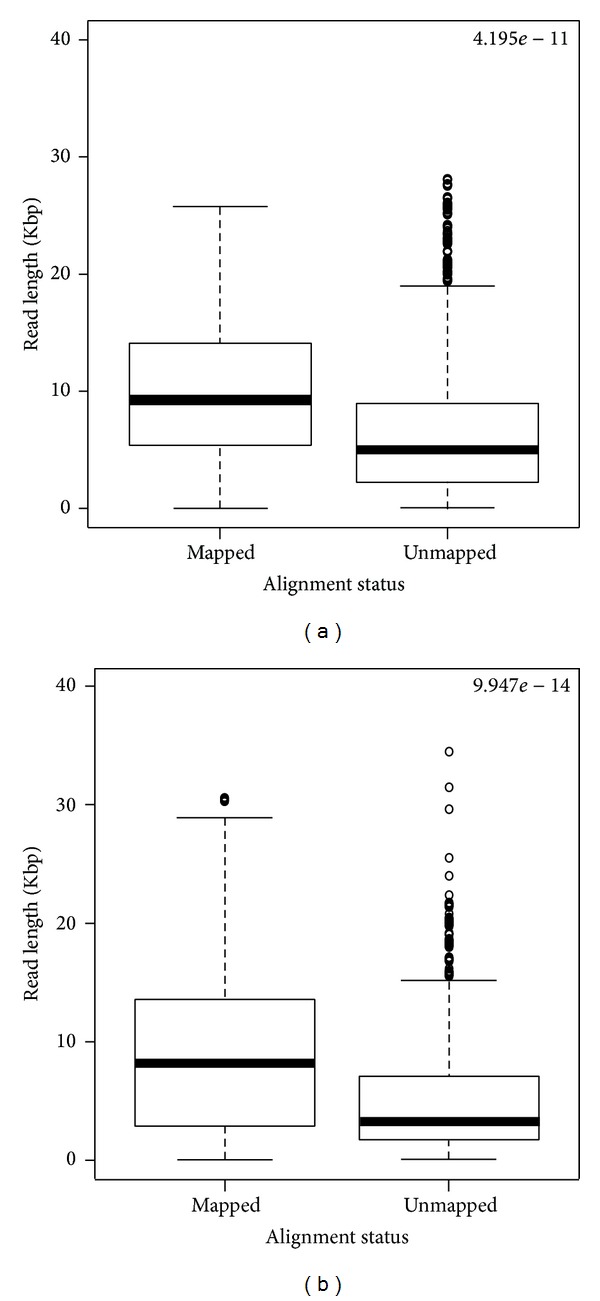
Length of mapped and unmapped reads for PacBio Human-Ref19-1 (a) and Human-Ref19-2 dataset (b) obtained with BWA-MEM. *P* value from two-tailed unpaired* t*-test is reported on top.

The same analysis was done on BLASR and BWA-MEM alignment results.  Figure 4 shows that boxplot for mapped and unmapped reads for both human PacBio datasets BLASR is the only one tool that is able to align the whole dataset.

We verified this observation also using BLASR and BWA-MEM tools. All reads were aligned using the former algorithm (data not shown), while BWA-MEM produced similar results of Bowtie2 algorithm in terms of distribution of reads length (Figure 4). In fact, we observed again a difference between mapped and unmapped reads with the latter characterized by a significantly shorter length.

*Error Correction. *In [41], statistics about errors in PacBio datasets are shown. It is reported that (i) the error rate that comes from sequencing is typically around 15%; (ii) errors are uniform within reads and not at the end (as seen with other sequencing technologies); and (iii) errors depend on insertions for the 11%, 4% on deletions and 1% on mismatches. Taking into account the possibility that results discussed above could be caused by errors related to the PacBio sequencing techniques, we tried to align a corrected dataset. For this test, we used the* Drosophila melanogaster* corrected dataset, generated by using the PacBio RS technology [42], and consisting in 332,369 reads with a median length of 1,186 bp.

**Figure 5:**
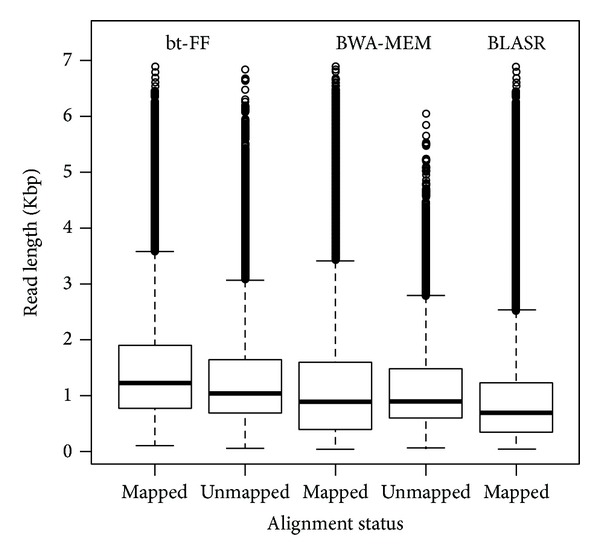
Length of mapped and unmapped reads for PacBio* Drosophila melanogaster* corrected dataset obtained by different alignment tools.

Notably, using Bowtie2, the fraction of aligned reads increased to 84.3% but again, we observed a fraction of unmapped reads whose length was significantly lower compared to the former (unpaired *t* test *P* value <2.2^−16^) (see Figure 5). The alignment of the same dataset was repeated with two well-known long reads alignment tools: BWA-MEM and BLASR. BLASR performed better on alignment rate with the whole dataset successfully aligned while BWA-MEM outperformed in processing time (sequential executions: 766 seconds for BWA-MEM, 7,668 seconds for bt-FF, 12,532 for BLASR, and 13,845 for bt-2.2.1).

## 5. Performance Comparison and Analysis

In this section, the original multithreaded implementation of Bowtie and BWA alignment tools are compared for performance to their porting onto the FastFlow pattern-based library on different datasets. A key of used tools with their version is reported in Table 3.

Tests were executed on an Intel workstation with 4 eight-core E7-4820 Nehalem (64 HyperThreads) @2.0 GHz with 18 MB L3 cache and 64 GBytes of main memory with Linux x86_64. Each processor uses HyperThreading with 2 contexts per core. bt-2.2.1 was compiled with the sse flag set to 4.2, while bt-2.0.6 and bt-FF have sse3 flag set. All were compiled with the g++ 4.7.2 compiler.

### 5.1. Bowtie2 on Roche 454 and Illumina Datasets (Mixed Read Length)

**Figure 6:**
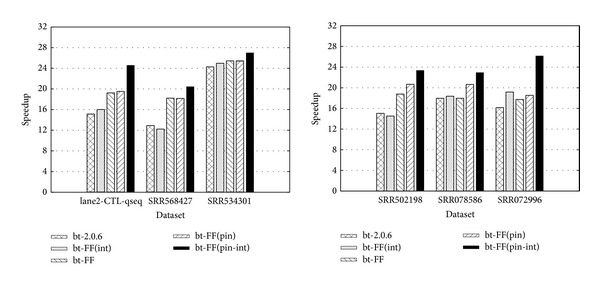
Maximum speedup obtained by executing different implementations of Bowtie2 on short reads datasets (see Table 1). The speedup is computed with respect to the bt-2.0.6 sequential execution.

As reported in Figure 6, bt-FF performs better than the original version (bt-2.0.6) on datasets with short reads, gaining up to 10 speedup points. In this case, the performance gain is mostly due to particularly low synchronisation overhead of the FastFlow run-time support, which is the main speedup-impairing factor on fine grain tasks.

Notice that the version with pinning and interleaving performs better in the most cases. This latter version is used for tests shown in the rest of the paper. Alignment tests with Roche 454 real and synthetic datasets can be found in [43], where Bowtie2 has been compared to other tools, reporting a poor speedup as well (maximum speedup of 7). Besides this, alignment algorithm is able to align very long reads, but its performance significantly degrades on long reads (whereas it is quite efficient on short reads).

**Figure 7:**
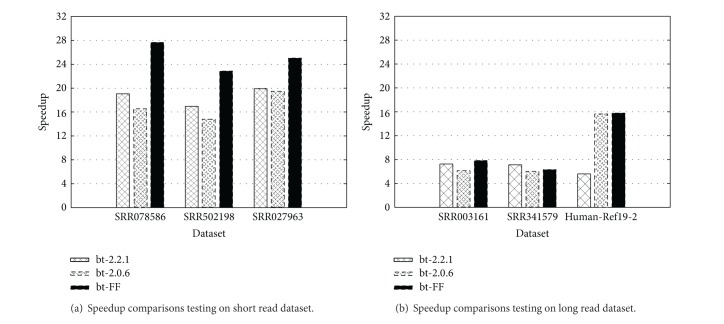
Speedup comparisons among bt-2.2.1 and bt-FF on mixed length datasets (see Table 1). The speedup is computed with respect to the bt-2.2.1 sequential execution.

As shown in Figures 7(a) and 7(b), bt-2.2.1 performs better than bt-2.0.6 but not better than the bt-FF. Apparently, the gain due to the SSE4.2 instructions in bt-2.1.1 (to perform character searches and comparison on two 16 bytes operands) on Nehalem platform does not compensate the limited speedup. Paradoxically enough, the wider SSE4.2 instructions induce even more pressure on memory subsystem, which is the real bottleneck of Bowtie2.

### 5.2. PacBio Human Datasets

Performances of bt-2.2.1, bt-2.0.6, and bt-FF have been also compared on two Human uncorrected datasets, which exhibits long length reads. Notice that the aim of the test is to assess the performance gain due to the high-level programming approach, that is, to compare bt-FF against bt-2.06 and bt-2.2.1, and not to assess absolute performance of Bowtie2 on long reads.

Table 4 shows execution times of all three versions tested on a small subset of 1000 reads of PacBio datasets, which alignment rate is shown in Figure 3 (Human-Ref19-1 and Human-Ref19-2). In this case, the performances of bt-2.0.6 and bt-FF are almost identical. On long reads, the mapping of a single read by means of the Bowtie2 algorithm becomes particularly expensive. In terms of concurrency, this turns into coarse grain tasks dispatched to the workers and thus into the reduction of speedup difference between bt-2.0.6 and bt-FF. It can be noticed that bt-2.2.1 is significantly slower than bt-2.0.6 when executed on PacBio datasets.

### 5.3. PacBio* Drosophila melanogaster* Dataset

To exclude the influence of sequencing errors content in sequences, we compared the speedup achieved by different tools (Bowtie2, BWA, and BLASR) on the PacBio* Drosophila melanogaster* corrected dataset.

**Figure 8:**
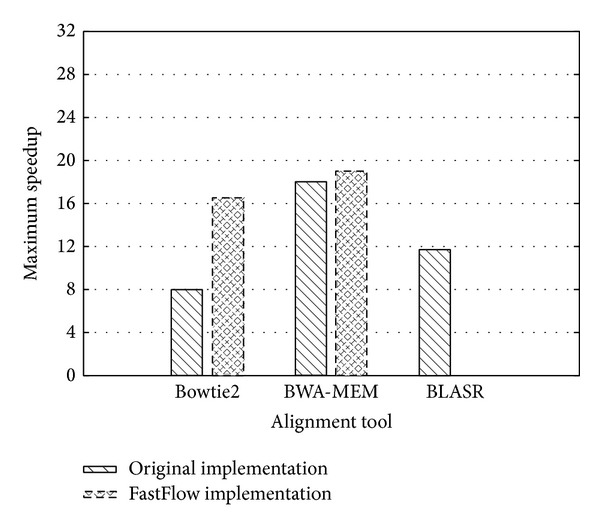
Performance comparison among bt-2.2.1, bt-FF, BLASR, BWA-MEM, and BWA-MEM-FF on* Drosophila melanogaster* corrected dataset. Speedups are computed with respect to the sequential execution of each tool.

Figure 8 shows the maximum speedup reached by Bowtie2 and BWA, in both the original version and the FastFlow versions. In all tested cases, the FastFlow version exhibits a better speedup with respect to the original, hand-optimised code. For Bowtie2, the gain is particularly consistent (2×), whereas BWA results only slightly improved. The rationale of this difference is likely to be related on the different memory behaviour of the two tools. Bowtie2 is strongly memory bound and can greatly benefit from affinity scheduling and shared data interleaving (enabling the usage of the aggregated bandwidth of memory channels) offered by FastFlow master-worker, whereas BWA exhibits a smaller working set and thus better exploits memory cache hierarchy.

The BLASR version, for which a FastFlow version has not been developed, is reported for the sake of completeness. BLASR exhibits the very same parallel structure of other two tools (see Section 3.2) and its porting to FastFlow is expected to be neither problematic nor very informative. In terms of absolute performances, the BWA tool on this dataset is order of magnitudes faster than Bowtie2, which is not designed to efficiently map long length reads.

In all cases, the efficiency of parallelisation hardly reaches the 50% of efficiency, which cannot be considered to be fully satisfactory. A full explanation for this result requires further investigation; however, it is likely related to SIMD/SSE code used for the single read mapping. SSE instruction typically induce a strong pressure on memory subsystem due to wide operands, thus saturating memory/shared-cache bandwidth even using the half of the available cores. A partial evidence of this effect can be found in Table 5: due to wide SSE4.2/STTNI instructions, bt-2.2.1 exhibits a much lower IPC (instruction per cycle) with respect to other versions of the tools coded with SSE2.

### 5.4. Performance Analysis

Further information to explain performance differences of the different versions of Bowtie2 can be extracted via perf, a performance analyser tool in Linux. Table 5 reports the performance analysis on Human-Ref19-1 dataset restricted to reads of length ranging from 2 Kbp to 3 Kbp. Results are obtained by way of the “perf stat –d” command. All the three versions of the tool have been analysed, running with 32 threads. These ranges are chosen on the basis of lower alignment rate reported on Table 2.

Table 5 shows that the number of threads migration by pinning worker threads in bt-FF version is strongly lower than the other two versions. The main thread is not pinned and then those migrations could be imputable to the main thread.

### 5.5. Testing on an Alternative Platform

**Figure 9:**
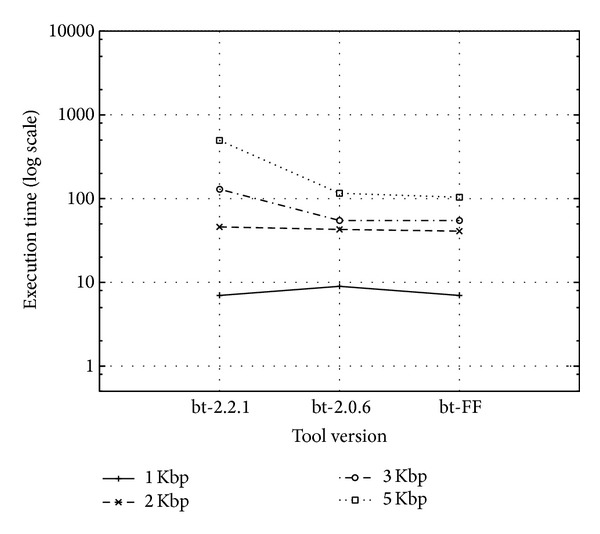
Execution time for each tool version (bt-2.2.1, bt-2.0.6, and bt-FF) on tested PacBio human subdatasets on the Intel Nehalem workstation.

**Figure 10:**
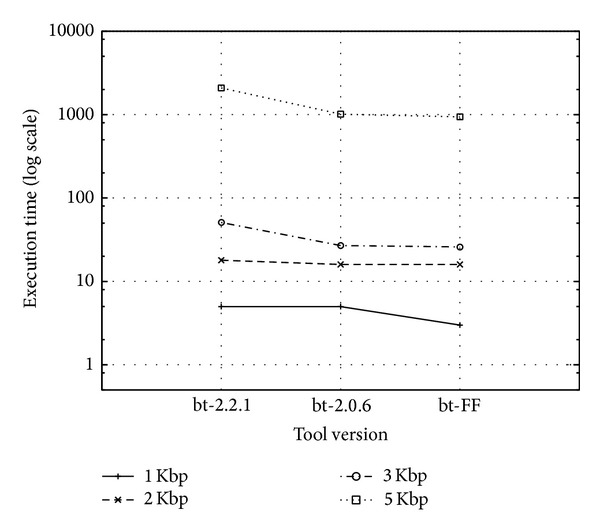
Execution time for each tool version (bt-2.2.1, bt-2.0.6, and bt-FF) on tested PacBio sub-datasets on the Intel Sandy Bridge workstation.

To assess results across different platforms, the tools were tested also on a different platform, an Intel Sandy Bridge with two 8-core sockets (2 HyperThreads) @2.2 GHz, 20 MB L3 cache with Linux x86_64 (only on a subdataset from Human-Ref19-1 from 1 Kbp up to 5 Kbp). As in the previous tests, bt-2.2.1 was compiled with the sse flag set to 4.2, while bt-2.0.6 and bt-FF have the sse3 flag set. All were compiled with g++ 4.4.7 compiler (4.7.2 for the Nehalem Workstation). Tests are executed by running tools with at most 16 threads in order to exploit only physical cores. These tests showed the same behaviour among the three versions, with two noteworthy differences: (1) all the Bowtie versions are significantly faster on the Sandy Bridge platform on subdatasets 1 Kbp, 2 Kbp, and 3 Kbp; (2) this is not true on subdataset 5 Kbp, for which the bt-2.2.1 version is also significantly slower with respect to the other software versions. Both differences are rooted in coupling of the software implementation choices with hardware platforms and can be appreciated by comparing Figures 9 and 10, which show completion times on subdatasets in four different ranges min—1 Kbp, min—2 Kbp, min—3 Kbp, and min—5 Kbp, where min is the minimum read length in the dataset. Being one subset of the next, respectively, they exhibit a growing size.

In general, the Sandy Bridge platform is (slightly) highly clocked and more importantly exploits SSE/AVX instructions whose length is twice the Nehalem's SSE ones. However, the quad-socket Nehalem platform exhibits an aggregate Level3 cache of 72 MB (18 MB x 4), whereas the Sandy Bridge dual-socket is only 40 MB (20 MB x 2). For this, the 5 Kbp experiment working set fits in the Nehalem cache and does not fit in the Sandy Bridge cache. Being Bowtie, a strongly memory-bound application, this impairs the performance to such large degree that cannot be balanced by the faster processors of the Sandy Bridge. In the same way, bt-2.2.1 is generally slower with respect to bt-2.0.6/bt-FF on the same experiment because it requires a larger working set.

## 6. Conclusions

In this paper, we analysed the problem of sequence alignment from parallel computing perspective; we reviewed the design of three of the most popular alignment tools exhibiting parallel computing capabilities, among others, Bowtie2, BWA, and BLASR. All these tools exploit a master-worker parallel orchestration paradigm to process the set of reads in parallel. Some of them also exploit SIMD parallelism to further accelerate the computation of a single task (i.e., a read) using SSE instructions. Each of the analysed tools implements its own version of the master-worker paradigm at a very low-level of abstraction, specifically using blocking locks of the Posix Threads library or processor-specific atomic instructions.

We advocate high-level parallel programming as an alternative design strategy for next generation alignment tools. High-level parallel programming aims at reducing development and performance tuning effort and enhances code and performance portability across different platforms. We demonstrated on two tools (Bowtie2 and BWA-MEM) that the pattern-based design not only simplifies tool engineering but also boosts the speedup of the application beyond the hand-tuned low-level original code. As nowadays no developer expects to get any performance advantage coding an application in assembler, no developer should expect to get more speedup by the low-level coding of a parallel application.

We ported Bowtie2 and BWA on top of the pattern-based FastFlow parallel programming framework for C++. The porting required altering few lines of code (out of several ten thousands) with an estimated programming effort of few days. Also, the FastFlow-based version of the tools resulted easier to tune for maximum performance. In particular, scheduling policy, load-balancing strategies, and memory affinity are extrafunctional features of the master-worker FastFlow pattern. Leveraging on these features, it has been possible to optimise tools parallel behaviour beyond the hand-optimised code of their original versions. As an example, in the case of Bowtie2, which is a memory bound application; the key optimisation consists in improving locality of the memory accesses and utilisation of shared memory bandwidth. In terms of programming effort, this just consists in configuring the master-worker pattern to adopt a memory-affine task scheduling.

High-level parallel programming is becoming the mainstream approach for a growing class of applications. Even though our results cannot be considered fully demonstrative of the correctness and efficiency of the parallel pattern applied, we can fairly state that the global structure of an aligner, from the parallelisation viewpoint, can be always mapped within a master-worker pattern with suggested optimisations. We do believe this can be an enabling feature for future generation sequence alignment and analysis approaches.

## Figures and Tables

**Table 1:** Datasets.

Dataset	Platform	Read length (bp)	Reads count
SRR534301	Illumina	101	108,749,331
SRR072996	Illumina	20	60,673,318
lane2_CTL_qseq	Illumina	36	53,673,423
SRR568427	Illumina	36	53,594,954
SRR502198	Illumina	36	25,675,656
SRR078586	Illumina	8–48	3,101,013
SRR003161	454 GS FLX	47–4,931	1,376,701
SRR341579	Illumina	202	6,143,624
SRR027963	Illumina	76	18,145,940
Human-Ref19-1	PacBio	35–35,488	25,249
Human-Ref19-2	PacBio	35–34,583	17,797
Drosophila M.	PacBio	55–6,883	332,369

**Table 2:** Alignment percentages on Human-Ref19-1 and Human-Ref19-2.

Subset	Mapped	Number of reads	Mapped	Number of reads
	PacBio Human-Ref19-1		PacBio Human-Ref19-2	
1 Kbp	8.10%	2098	10.76%	1747
2 Kbp	8.20%	5231	11.68%	4788
3 Kbp	9.57%	2697	12.07%	7093
5 Kbp	9.58%	11755	13.38%	9931

**Table 3:** Alignment tools key.

Acronym	Tool	Version	Variant	Technology
bt-2.0.6	Bowtie2	2.0.6	original	Pthreads
bt-2.2.1	Bowtie2	2.2.1	original	Pthreads
bt-FF	Bowtie2	2.2.1	porting	FastFlow
BWA-MEM	BWA MEM	0.7.9a	original	Pthreads
BWA-MEM-FF	BWA MEM	0.7.9a	porting	FastFlow
BLASR	BLASR	2.1	original	Pthreads

**Table 4:** Bt-2.2.1, Bt-2.0.6, and Bt-FF execution times on Human-Ref19-1 and Human-Ref19-2.

Tool	Best time	Speedup	Best time	Speedup
	PacBio Human-Ref19-1		PacBio Human-Ref19-2	
bt-2.2.1	00:14:48	6.34	00:20:38	5.6
bt-2.0.6	00:02:20	16.30	00:03:15	15.61
bt-FF	00:02:20	16.30	00:03:13	15.77

**Table 5:** *Perf Stat* analysis on Human-Ref19-1, 2-3 Kbp for different implementations of Bowtie2.

Metric	bt-2.2.1	bt-2.0.6	bt-FF
CPUs utilised	28.665	19.661	24.363
CPU-migrations	1,363	3,513	57
**IPC**	0.19	0.98	1.03
**L1-dcache-misses**	42.46%	32.91%	32.14%
(of all L1-dcache hits)			
**LLC-load-misses**	80.87%	58.66%	67.91%
(of all LL-cache hits)			
Execution time (s)	96.87	24.41	19.05
